# Natural History of Isolated Below-Knee Vein Thrombosis in Patients with Spinal Cord Injury

**DOI:** 10.3390/healthcare9070861

**Published:** 2021-07-08

**Authors:** Jang-Hyuk Cho, Dong-Gyu Lee

**Affiliations:** 1 Department of Physical Medicine, Keimyung University Dongsan Hospital, School of Medicine, Keimyung University, Daegu 42601, Korea; rehacho@hanmail.net; 2 Department of Physical Medicine and Rehabilitation, College of Medicine, Yeungnam University, Daegu 42415, Korea

**Keywords:** spinal cord injury, deep vein thrombosis, anticoagulation, distal deep vein thrombosis, surveillance

## Abstract

In the general population, serial imaging is recommended over anticoagulant therapy for below-knee deep vein thrombosis (BKDVT). However, no clinical trial in Asian patients with spinal cord injury and BKDVT has been performed. Therefore, we evaluated the natural course of BKDVT in patients with acute spinal cord injury. We retrospectively analyzed inpatients with spinal cord injury with BKDVT between 2016 and 2020. All patients underwent inpatient rehabilitation treatment and duplex ultrasonographic examination of both the lower extremities at follow-up. After screening 172 patients with acute spinal cord injury for deep vein thrombosis using duplex ultrasound, 27 patients with below-the-knee deep vein thrombosis were included in this study. The mean lower-extremity motor score (median, interquartile range) was 66.0, 54.0–74.5. Sixteen patients received a non-vitamin K antagonist oral anticoagulant (NOAC) for anticoagulation. None of the patients had proximal propagation according to the follow-up duplex ultrasonography. BKDVT disappearance was not significantly different between the NOAC treatment and non-treatment groups. Asian patients with spinal cord injury have a low incidence of venous thromboembolism and favorable natural history of BKDVT. We recommend serial imaging over anticoagulant therapy for BKDVT in these patients.

## 1. Introduction

Patients with spinal cord injury have a high morbidity, including deep vein thrombosis [1]. Spinal cord injury is associated with high postoperative coagulability, immobility, trauma-induced venous endothelial injury, and high infection rates [2]. Pulmonary embolism is a critical and devastating event in patients with spial cord injury. Although deep vein thrombosis and pulmonary embolism are not always coincidental, deep vein thrombosis is one of the predisposing factors for pulmonary embolism [3]. Therefore, surveillance and treatment of DVT in patients with spinal cord injury is an essential strategy in preventing pulmonary embolism.

Given the high morbidity and mortality due to venous thromboembolism in spinal cord injury, thromboprophylaxis has been one of the principal treatments for patients with spinal cord injury [1]. The evidence on thromboprophylaxis for acute spinal cord injury is mostly based on studies conducted in Western patients with spinal cord injury. However, Asian populations have a relatively low risk of venous thromboembolism than their Western counterparts [4,5]. Asian patients with spinal cord injury have also been shown to have a relatively low risk of deep vein thrombosis [6]. The incidence of gastrointestinal bleeding has been reported to be 5–22% in patients with spinal cord injury [7]. Moreover, 5.2% of the patients with spinal cord injury experience serious bleeding, resulting in changes in vital signs and requiring transfusions. Because of the adverse effects of thromboprophylaxis and low incidence of venous thromboembolism, preventive thromboprophylaxis has not been established as a standard treatment in Asian patients with acute spinal cord injury [8].

The treatment guidelines for BKDVT recommend serial imaging for 2 weeks rather than anticoagulant therapy for isolated below-knee deep vein thrombosis (BKDVT). However, if such a patient has severe symptoms or risk factors, anticoagulant therapy is recommended for BKDVT [9]. However, these guidelines are based on weak findings and low-quality evidence. Moreover, no clinical trial or retrospective study on BKDVT in patients with spinal cord injury and on the incidence of proximal propagation in BKDVT has been conducted.

Therefore, in this study, we retrospectively evaluated the significance of BKDVT in patients with spinal cord injury with or without thromboprophylaxis based on follow-up duplex ultrasonographic assessments.

## 2. Materials and Methods

### 2.1. Study Participants

Data of patients with spinal cord injury with BKDVT between 2016 and 2020 were retrospectively collected. This study was approved by the Institutional Review Board of Yeungnam University Medical Center (protocol code; 2021-04-001) and the need for written informed consent from patients was waived. This study fully conformed to the Strengthening the Reporting of Observational Studies in Epidemiology Statement. The inclusion criteria were as follows: (a) spinal cord injury, (b) BKDVT diagnosis from duplex ultrasonography, and (c) follow-up duplex ultrasonographic assessment.

Although the global guidelines for acute spinal cord injury patients recommend routine anticoagulant thromboprophylaxis to reduce venous thromboembolism [10], South Korean medical societies do not have such guidelines. Therefore, around 2018, our hospital adopted short-term follow-up duplex ultrasonographic examinations instead of anticoagulant therapy in BKDVT according to the guidelines of the American College of Chest Physicians [11]. All inpatients with spinal cord injury routinely underwent rehabilitation therapy consisting of physical therapy, functional electrical stimulation, compression stocking application, and tilt table standing training.

Therefore, our patients with spinal cord injury had not received thromboprophylaxis. Duplex ultrasonographic assessments were routinely conducted in patients with spinal cord injury with D-dimer levels > 0.5 µg/mL as a screening test for deep vein thrombosis. Expert physicians examined both the lower limbs using a duplex ultrasound system (LOGIQ E9; GE Healthcare, Chicago, IL, USA). BKDVT was defined as deep vein thrombosis of the leg distal to the popliteal vein [12]. Proximal propagation and disappearance of BKDVT were observed during follow-up duplex ultrasonography.

To evaluate the natural course of BKDVT in spinal cord injury, patients were divided into two groups: the non-vitamin K antagonist oral anticoagulant (NOAC) group and the non-NOAC treatment group. Rivaroxaban (Xarelto; Bayer AG Pharmaceuticals, Berlin, Germany), an NOAC, was prescribed as the anticoagulant.

The lower-extremity motor score was evaluated according to the guidelines of the American Spinal Injury Association [13]. The lower-extremity motor score was calculated as the sum of the manual muscle testing scores for each of the five lower-extremity muscle groups: hip flexors, knee extensors, ankle dorsiflexors, long toe extensors, and ankle plantar flexors. Manual muscle testing uses a 6-point scale from 0 to 5. Therefore, the maximal point of the lower-extremity motor score was 50. The lower-extremity motor score was used to analyze the relationship between immobility and BKDVT propagation. Patients with spinal cord injury were divided into walker and sitter groups. Patients who were able to independently walk indoors or outdoors were allocated to the walker group, and those who could not independently walk even with equipment were allocated to the sitter group. The effect of ambulation on deep vein thrombosis propagation was evaluated by the allocation of the patients to the two groups.

The American Spinal Injury Association Impairment Scale is a classification scale used to classify the severity of spinal cord injury. The American Spinal Injury Association Impairment Scale is based on a 5-point scale, ranging from A (complete sensory and motor injury) to E (normal sensory and motor function) [14]. Patients with grade B have some sensory function without motor function. If motor grade of half of the key muscles below injury level is less than 3, the grade is Grade C. If at least half of the key muscles below the injury level have a muscle grade of 3 or more, then the grade is Grade D.

### 2.2. Statistical Analyses

Data input and statistical calculations were performed using SPSS ver. 25.0 (SPSS Inc., Chicago, IL, USA). Age and the lower-extremity motor score demonstrated normality in the Shapiro–Wilk test. Therefore, Student’s *t*-test was used to analyze differences in these factors between the groups. The chi-square test and Fisher’s exact test were applied to compare categorical factors between the non-NOAC and NOAC groups: follow-up duplex ultrasonography outcomes, ambulation, operation, and trauma. Statistical significance was set at *p* < 0.05.

## 3. Results

A total of 167 patients with acute spinal cord injury were examined using duplex ultrasonography from 2016 to 2020. In these, deep vein thrombosis was not observed in 107 patients (64%) on duplex ultrasonograms. Sixty patients (36%) had deep vein thrombosis within the lower extremities. Among these, 18 (11%) and 42 (25%) patients had deep vein thrombosis in the popliteal and/or thigh veins and below the knee, respectively. Of the patients with BKDVT, 15 (9%) had not been examined using duplex ultrasonography at follow-up. These patients were treated with an anticoagulant. Finally, 27 patients with spinal cord injury (19 men and 8 women) were included in this study (Table 1). The median and interquartile range of age was 64.0 years and 54.0–74.5. The median and interquartile range of lower-extremity motor score was 28.0 and 11.0–32.0. Twenty-two patients (81.5%) could not walk, and five patients (18.5%) could walk. Sixteen patients received anticoagulant therapy with an NOAC. Eleven patients were examined at follow-up using duplex ultrasonography without anticoagulant treatment. Initial duplex ultrasonography revealed unilateral BKDVT in 14 patients and bilateral BKDVT in 13 patients. The median and interquartile range of interval between the initial and follow-up duplex ultrasonographic examinations were 17.0 days and 10.0–23.5.

**Figure 1:**
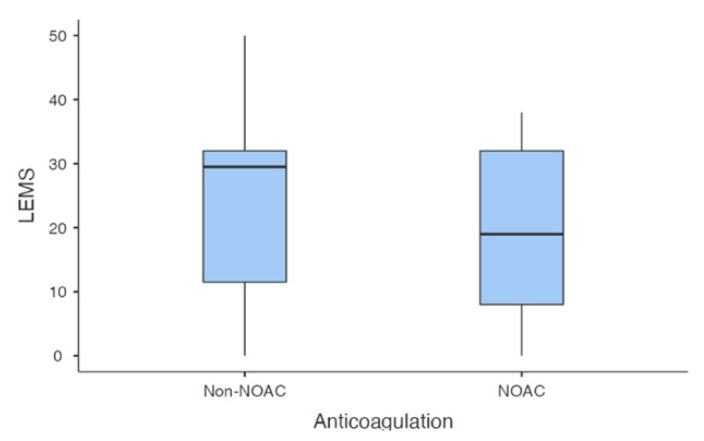
The lower-extremity motor score in the non-NOAC and NOAC treatment groups. There was no statistically significant difference between the two groups. NOAC, non-vitamin K antagonist oral anticoagulant.

The follow-up duplex ultrasonography did not reveal proximal propagation of BKDVT in any of the patients. Moreover, disappearance of the BKDVT was observed in five patients with spinal cord injury (31.3%) in the non-NOAC treatment group based on follow-up duplex ultrasonographic assessment (Table 2). BKDVT disappearance was not significantly different between the NOAC and non-NOAC treatment groups. The two groups were also not significantly different in terms of age, lower-extremity motor score, laterality, trauma, operation, and ambulation (Table 2 and Figure 1).

## 4. Discussion

Irrespective of receiving anticoagulant therapy, proximal propagation of BKDVT was not observed in any of the 27 patients with spinal cord injury. Although there was no significant difference in the clinical characteristics and risk factors for deep vein thrombosis, some patients in the non-NOAC treatment group demonstrated BKDVT disappearance and no propagation at the follow-up duplex ultrasonography, which was not significantly different based on NOAC treatment.

Pulmonary embolism is a devastating event in patients with acute spinal cord injury. The incidence of deep vein thrombosis in Western patients with spinal cord injury has been reported to be 62% [15]. Even with thromboprophylaxis, the incidence of deep vein thrombosis has been reported to range from 8.5% to 21.7% in patients with acute spinal cord injury [16]. Therefore, thromboprophylaxis is recommended to reduce deep vein thrombosis and/or pulmonary embolism. However, Asian-American and Asian populations have a lower incidence of venous thromboembolism than Western populations [4,17]. Our results also demonstrated a low risk of deep vein thrombosis in patients with spinal cord injury. Of the acute spinal cord injury patients screened for this study, 64% did not have deep vein thrombosis on duplex ultrasound. Only 11% of the patients had deep vein thrombosis in the proximal vein, which did not present any symptoms. Therefore, it needs to be elucidated whether the benefit of thromboprophylaxis in Asian patients with spinal cord injury outweighs its risks.

The clinical impact of deep vein thrombosis is because of proximal propagation resulting in pulmonary embolism. A low incidence of proximal propagation (5%) has been reported in inpatients with BKDVT. However, veins below the knee have a small diameter and low risk of proximal propagation [18]. In addition, BKDVT sometimes spontaneously resolves without anticoagulation treatment [19]. The clinical impact of BKDVT remains controversial because its association with pulmonary embolism has not yet been well established. Therefore, BKDVT treatment is equipoised for serial imaging and anticoagulation. However, to the best of our knowledge, there is no relevant study on the incidence of proximal propagation of BKDVT in Asian patients with spinal cord injury. Our study demonstrated that BKDVT in Asian patients with spinal cord injury has a relatively low clinical impact.

We speculated that this low risk of BKDVT propagation in patients with spinal cord injury may be attributable to race and the availability of a public health insurance system. As previously mentioned, Asians have a low risk of venous thromboembolism in the general population. The incidence of deep vein thrombosis in Korean patients with acute spinal cord injury has been reported to be 27.6% [6]. In this study, 21.1% of patients had an isolated distal deep vein thrombosis. Only 6.5% of the patients with acute spinal cord injury had a proximal deep vein thrombosis. Moreover, no fatal pulmonary embolism cases had occurred. The clinical impact of thromboprophylaxis in patients with acute spinal cord injury has been reported in Western populations. This result can be explained by the high incidence and mortality of pulmonary embolism in Western patients with spinal cord injury. Therefore, the clinical practice guidelines for acute spinal cord injury recommend anticoagulant thromboprophylaxis to minimize the risk of venous thromboembolism [1]. Considering the low incidence of fatal pulmonary embolism and deep vein thrombosis in Asian patients with spinal cord injury, anticoagulant thromboprophylaxis can be flexibly applied to Asian patients with spinal cord injury.

South Korea has an active rehabilitation system for patients with spinal cord injury. The public insurance system supports intensive rehabilitation and regulates the hospitalization period [20]. As a result, hospitalization duration at referral hospitals was reduced by approximately 25% compared with that reported previously. Hence, patients can receive early and well-organized inpatient rehabilitation treatments. We speculate that these factors also contribute to the favorable course of BKDVT in patients with acute spinal cord injury.

Gastrointestinal ulceration and bleeding are common comorbidities of acute spinal cord injury. The incidence of gastrointestinal bleeding has been reported to be 5.5% in acute spinal cord injury [21]. Fatal gastrointestinal bleeding has been reported in 2.5% of patients [22]. Cervical-level cord injury, trauma, infection, and high-dose steroid treatment increase the risk of gastrointestinal bleeding and major complications [23]. These are common factors in inpatients with acute spinal cord injury. Gastrointestinal complications are not only a frequent cause of rehospitalization but also a challenge in diagnosis [24,25]. Moreover, anticoagulant therapy increases the risk of gastrointestinal bleeding [26]. Therefore, thromboprophylaxis can be withheld in Asian patients with acute spinal cord injury with a high risk of gastrointestinal bleeding.

Motor weakness is generally known as the critical factor for deep vein thrombosis. Therefore, we analyzed whether there was a difference in the outcomes of BKDVT based on the NOAC treatment. The lower-extremity motor score and ambulatory function were not significantly affected by the outcomes of BKDVT. However, we were unable to conclude whether lower extremity weakness affects the prognosis of BKDVT. Although 81% of the patients were unable to walk independently, only seven patients had complete motor weakness, grade A and B of the American Spinal Injury Association Impairment Scale. Moreover, 74% of patients were able to move their lower extremities on the floor or in bed. Therefore, further study is required about the natural course of BKDVT for complete spinal cord injury patients.

This study had some limitations. First, it had a small sample size. We had initially screened 172 patients with spinal cord injury. The small sample size was partly because of the low incidence of venous thromboembolism in Asian populations. Therefore, a multicenter study is required to increase the sample size. Second, there was no evaluation for pulmonary embolism. Pulmonary embolism can occur without deep vein thrombosis [27]. We did not perform a routine computed tomography scan for pulmonary embolism screening in patients with BKDVT. Therefore, whether pulmonary embolism did not occur in patients with BKDVT could not be substantiated. However, in this study, patients with BKDVT were not diagnosed with pulmonary embolism.

## 5. Conclusions

Asian patients with spinal cord injury have a low incidence of venous thromboembolism and a favorable natural course of BKDVT. Therefore, a flexible strategy for deep vein thrombosis can be implemented in these patients. Finally, further studies are required to evaluate the usefulness of thromboprophylaxis in Asian patients with acute spinal cord injury.

## Figures and Tables

**Table 1:** Demographic data.

Variable	Data
Age (Median, interquartile range)	66.0, 54.0–74.5
lower-extremity motor score (Median, interquartile range)	28.0, 11.0–32.0
Sex (Male: Female)	19:8
Ambulation (No. of patients) Sitter: Walker	22:5
Cause of injury (No. of patients)	
Traumatic	12
Non-traumatic	
Spondylotic myelopathy	12
Cord infarct	1
Metastasis	2
Initial Duplex ultrasound (No. of patients, %)	
Unilateral: Bilateral	14(51.9%): 13(48.1%)
Follow-up duplex ultrasound (No. of patients) Non-DVT: DVT	11:16
Interval (day) between duplex ultrasound (median, interquartile range)	17.0, 10.0–23.5
The American Spinal Injury Association Impairment Scale (No. of patients, %)	
A	1 (3.7%)
B	6 (22.3%)
C	10 (37.0%)
D	10 (37.0%)

DVT, deep vein thrombosis.

**Table 2:** Differences between anticoagulation groups.

	Non–NOAC Group	NOAC Group	*p*-Value
Number	16	11	
The American Spinal Injury Association Impairment Scale			
(No. of patients, %)			
A	0	1	
B	6	0	
C	4	6	
D	6	4	
Age (median, interquartile range)	68, 60–68	63, 50.5–67.5	0.25
Male and Female (No. of patients)	12:4	7:4	0.675
lower-extremity motor score (median, interquartile range)	29.5, 11.5–32.0	19, 8.0–32.0	0.40
Ambulation (No. of patients)			
Sitter vs. walker	13:3	9:2	0.97
Initial duplex ultrasound (No. of patients)			
Unilateral:Bilateral	10:6	4:7	0.18
Trauma vs. Non-trauma (No. of patients)	8:8	4:7	0.69
Operation vs. Non-operation (No. of patients)	12:4	10:1	0.61
Follow-up Duplex ultrasound			
(No. of patients)			
No DVT vs. DVT	5:11	6:5	
Proximal propagation	0	0	0.23

DVT, deep vein thrombosis.

## Data Availability

The data presented in this study are available upon request from the corresponding author.
